# Investigation on Applying Cyclodextrins in a Fermentation Process for Enhanced Biosurfactant Production by *Bacillus licheniformis*

**DOI:** 10.3390/ijms262110518

**Published:** 2025-10-29

**Authors:** Jesse John Sakiyo, Áron Németh

**Affiliations:** Department of Applied Biotechnology and Food Science, Faculty of Chemical Technology and Biotechnology, Budapest University of Technology and Economics, Műegyetem rkp. 3, 1111 Budapest, Hungary; jsakiyo@edu.bme.hu

**Keywords:** *Bacillus licheniformis*, lichenysin, alpha-cyclodextrin, beta-cyclodextrin, gamma-cyclodextrin, DIMEB

## Abstract

Biosurfactants are environmentally friendly alternatives for chemical surfactants and have a broad spectrum of applications in different industries such as cosmetics, oleochemistry, pharmaceuticals, and detergents. It has been established that *Bacillus licheniformis* produces several lipopeptide-type biosurfactants, including lichenysin and iturin. However, in order to enhance the biosurfactant production by *Bacillus licheniformis*, it is necessary to either extend the already performed media optimization to circumvent the current limitations or defeat the product inhibition. Cyclic oligosaccharides made of glucose monomers called cyclodextrins (CD) have been shown to improve the biomass synthesis of other microorganisms, which may also increase the output of biosurfactants. The efficient fermentative production of biosurfactants is often limited by the inhibitory/toxic effect of the product on the producer cells itself. Therefore, in this work, we demonstrated that CDs may entrap biosurfactants from the broth, decreasing product inhibition. Thus, we also tested the media supplementation with three different types of cyclodextrins including alpha-, beta-, and gamma-CD and a derivative (dimethyl-beta-cyclodextrin, DIMEB); notably, DIMEB at 2.0 g/L enhanced biosurfactant production by up to 41.43% and specific product formation (g product/g cells) by 79,6% compared to the control, while mitigating the growth inhibition observed at lower concentrations. This study demonstrates, for the first time, the distinct advantage of DIMEB over native CDs in reducing product toxicity and boosting biosurfactant yields, highlighting its potential as a simple additive strategy for improving sustainable bioprocesses.

## 1. Introduction

As concern for sustainability and eco-friendliness has increased in recent years, there has been a significant focus on researching alternative surfactants across industries [1,2]. Biosurfactants, characterized by their amphiphilic nature, which combine hydrophilic and hydrophobic constituents within their molecular structure, represent a promising area of research. The hydrophilic portion typically consists of carbohydrate, peptide, or amino acids, whereas the hydrophobic part is generally a fatty acid or hydrocarbon chain. Due to the amphiphilic nature of biosurfactants, they can interact with both polar and nonpolar substances, resulting in their unique surface-active properties [3,4,5]. Biosurfactants, generated by various microorganisms, exhibit exceptional surface-active properties and have many applications, including enhanced oil recovery, bioremediation, and pharmaceutical formulations [6,7,8].

*Bacillus licheniformis*, a Gram-positive, mesophilic, and soil-dwelling bacterium with diverse metabolic capabilities, has been identified as an effective biosurfactant manufacturer. It has been extensively studied for its capacity to produce lichenysin, a lipopeptide biosurfactant with a similar structure to surfactin, which is one of the most effective and best known biosurfactants produced by *Bacillis subtlis* [9,10]. Lichenysin, though structurally similar to surfactin, was selected for this study because of its stability and efficiency. It retains surface activity under high temperature, salinity, and extreme pH, making it valuable for industrial uses such as oil recovery and bioremediation. The reported critical micelle concentration (CMC) values for lichenysin are lower than for surfactin, indicating a higher efficiency in reducing the surface tension [11,12]. To optimize the biosurfactant production process and enhance its yield, several factors must be considered, including the influence of specific carbon sources on the biosynthetic efficiency of *Bacillus* spp. For example, the use of synthetic media supplemented with pure glycerol has been shown to significantly enhance the surfactin production in *Bacillus subtilis*, due to its high carbon content and ease of assimilation by microbial cells [13,14]

Additionally, another study demonstrated the effectiveness of *Beta vulgaris* waste, which varied in its agro-waste concentration, pH, and temperature, and was used in the production of biosurfactant by *Bacillus licheniformis* STK 01. This result showed a strong tendency for hydrocarbon emulsification and was able to reduce the surface tension down to 30 mN/m [15,16]. *Bacillus* spp. Lipopeptides’ biosurfactant products, such as surfactin, iturin, lichenysin, and fengycin, are widely studied families. The peptide moiety of the surfactin is synthesized using huge multienzymatic proteins called NonRibosomal Peptide Synthetases. This mechanism is responsible for the peptide biodiversity of the members of the surfactin family. In addition, on the fatty acid side, fifteen different isoforms (from C12 to C17) can be incorporated, increasing the number of the surfactin-like biomolecules [17,18,19].

Due to their extraordinary solubilizing properties, low toxicity, and minimal inflammatory response, cyclodextrins (CDs) have attracted considerable interest in pharmaceutical formulations [20,21] or in bioremediation processes. Composed of glucose units, these cyclic oligosaccharides have a unique truncated cone-shaped structure, which is ~0.79 nm in height, and whose inner cavity diameters are ~0.47–0.53 nm (α-CD), 0.60–0.65 nm (β-CD), and 0.75–0.83 nm (γ-CD) [22], which enables them to encapsulate smaller hydrophobic molecules. While the enhancement of microbial metabolism by CDs is not new, their applications in biosurfactant fermentations have not yet been studied. However, CDs can encapsulate hydrophobic lipopeptides moiety of the product lichenysin, lowering their free concentration in broth, reducing the direct membrane contact, and thus mitigating the toxicity to *Bacillus* cells. By forming inclusion complexes, CDs can also reduce the product’s feedback inhibition [20,22]. CDs are extensively used as excipients to improve the solubility, stability, and bioavailability of pharmaceuticals. Cyclodextrins have been proposed as a ‘greener’ alternative to organic solvents and synthetic surfactants for enhancing the bioavailability of organic compounds in soils. Cyclodextrins may form inclusion complexes with hydrophobic contaminants, boosting their water solubility and bioremediation in soils [23,24]. Recent research has uncovered an additional intriguing characteristic of CDs: their antimicrobial properties. A microwave-assisted Huisgen reaction may be used to react CDs with an amphiphilic substance resulting in CD derivatives that can function as membrane disruptors. The capacity of these derivatives to permeabilize bacterial membranes is dependent on the amino substituents and an adequate balance of hydrophobicity and hydrophilicity, allowing the production of derivatives with specific toxicity against bacteria [25,26,27,28].

No published study has investigated the use of CDs to mitigate lichenysin-associated product inhibition in Bacillus licheniformis. This work is the first to directly compare native CDs with a methylated derivative in this context. Our contribution lies in linking the CD type to its measurable effects on the biomass growth, glucose uptake, and biosurfactant yield. Since one of the major bottlenecks during biosurfactant yield improvement is that the product may destroy the producer strain’s membrane causing product inhibition [29,30], in this study, we aimed to test the potential positive effect of cyclodextrins through entrapping the product biosurfactant. CD’s entrapping capability is mostly defined by their internal pocket diameter; hence, we compared three different CDs and one potent derivative in terms of the biosurfactant fermentations time curves and of the biosurfactant amount achieved.

## 2. Results

Lichenysin, the major biosurfactant produced by *Bacillus licheniformis* belongs to secondary metabolites [31]; therefore, the fermentation starts with the *trophophase,* i.e., cell growth, followed by *idiophase,* i.e., product formation. The effect of media supplementation with different amounts and types of cyclodextrins is therefore investigated, compared, and evaluated for cell growth and product formation separately.

### 2.1. Cell Growth and Specific Growth Rate in a Reverse Spinning Personal Bioreactor (10 mL)

These small-scale experiments were applied to determine CD’s effect on cell growth from two aspects: regarding the specific growth rate and the final biomass amount after the *trophophase* (50 h) before the *idiophase*.

#### 2.1.1. Impact of Cyclodextrin Derivatives on Maximum Specific Growth Rate (Umax)

**Figure 1:**
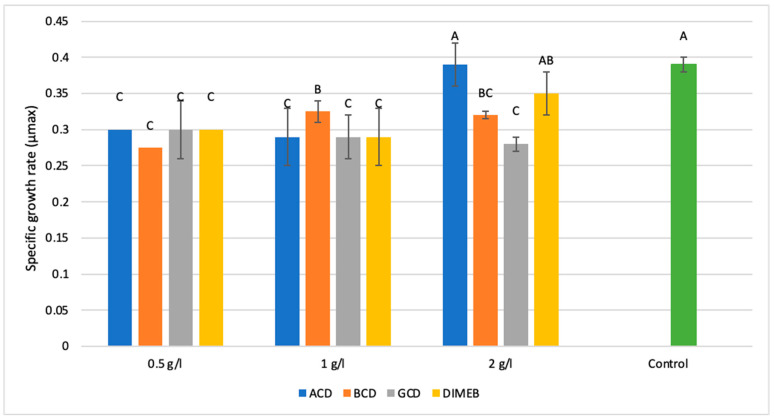
Specific growth rate (h^−1^), applying different cyclodextrins at 0.5, 1.0, and 2.0 g/L, compared to the untreated control (green bar). Bars with different letters (A–C) indicate statistically significant differences between treatments.

To assess the influence of cyclodextrin derivatives on the microbial growth kinetics, the maximum specific growth rate (Umax) was determined using data from a spinning personal bioreactor system. The treatments included α-cyclodextrin (ACD), β-cyclodextrin (BCD), γ-cyclodextrin (GCD), and dimethyl-β-cyclodextrin (DIMEB) at concentrations of 0.5, 1.0, and 2.0 g/L. The control group was grown without cyclodextrin supplementation. The results are summarized in Figure 1.

The addition of different cyclodextrins (CDs) to the fermentation medium had a clear concentration- and type-dependent inhibitory effect on the maximum specific growth rate (Umax) of *Bacillus licheniformis*, rather than an enhancing one. The control condition consistently showed the highest Umax values, while supplementation with CDs generally reduced the growth rates. At lower concentrations (0.5 and 1 g/L), α-CD, β-CD, γ-CD, and DIMEB produced broadly similar levels of growth inhibition showing 20–25% less growth rate compared to the control.

Interestingly, at the highest tested concentration (2 g/L), α-CD maintained a Umax similar to the control, suggesting no inhibitory effect, which contrasts with the general inhibitory concentration dose curve, since more supplementation results commonly in higher inhibition levels [32]. Additionally, these results already highlight the effect of methylation on β-CD: because DIMEB’s effect on the specific growth rate is rather similar to a α-CD’s effect, rather than t β-CD, this suggests that the methylation altered the cavity size (probably reduced) or the hydrophobic character.

On the other hand, β-CD showed consistently lower Umax values than the control at all tested concentrations similar to γ-CD. Statistical analysis confirmed that these results do not differ from each other significantly, indicating a generally uniform inhibitory effect across the tested range regarding this two CD type.

Taken together, the data show that under the tested conditions, CD supplementation acted more as a growth inhibitor than an enhancer, and the inhibitory effect could be defeated with higher (2 g/L) concentration of α-CD and DIMEB.

#### 2.1.2. Effect of Cyclodextrin Treatments on Biomass Concentration at 50 h

To evaluate the impact of various cyclodextrins and the derivative on microbial growth, the optical density at 850 nm (OD_850_) was compared at the 50th hour of cultivation. The 50 h time point was selected, because it is already in the stationary growth phase (i.e., the end of *trophophase*), while the personal bioreactor experiment focused mainly on determining the specific growth rate (Umax). Additionally, the fermentation volume at this scale was insufficient for downstream analyses such as product isolation or surface tension measurement; therefore, we did not wait for idiophase. The results, expressed as the mean of OD_850_, are shown in Table 1. A paired *t*-test was used to assess the statistical significance.

The analysis showed that, in most treatments and concentrations, cyclodextrin supplementation did not lead to statistically significant changes in OD_850_ when compared with the control group. However, two exceptions were observed: α-CD at 2 g/L and DIMEB at 1 g/L, both of which produced small but statistical significance; however, while the ACD increased, the DIMEB decreased the amount of biomass.

To summarize the cell-growth related results in the small-scale *trophophase* experiments, application of α-CD, b-CD, g-CD, and DIMEB in low concentrations of 0.5–1 g/L resulted both a slower growth rate but a non-significantly differing biomass amount (in other words, only the growth rate was reduced, the biomass amount not). However, at a higher concentration (2 g/L), α-CD did not slow down the growth rate and also increased the biomass amount raising interesting questions about the mechanism. DIMEB worked similar to α-CD.

### 2.2. Fermentation Analysis of Shaking Flasks (150 mL)

**Figure 2:**
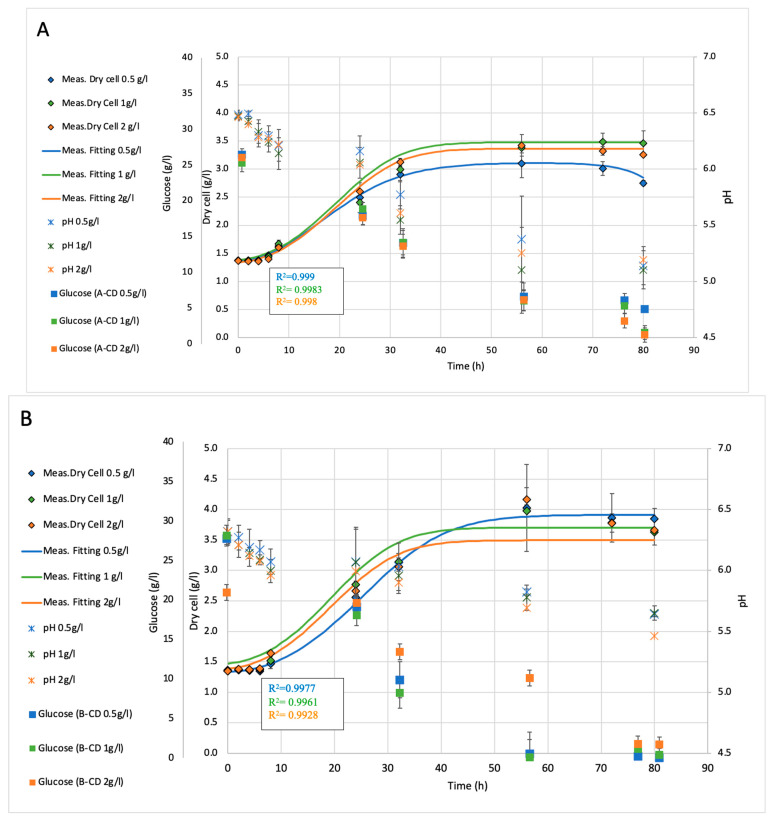

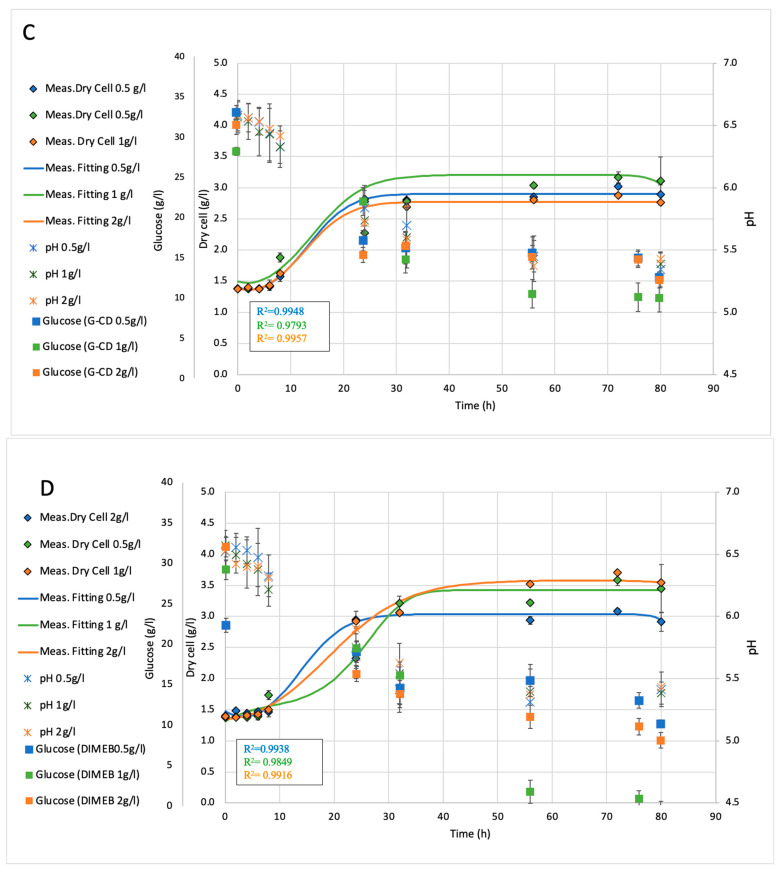

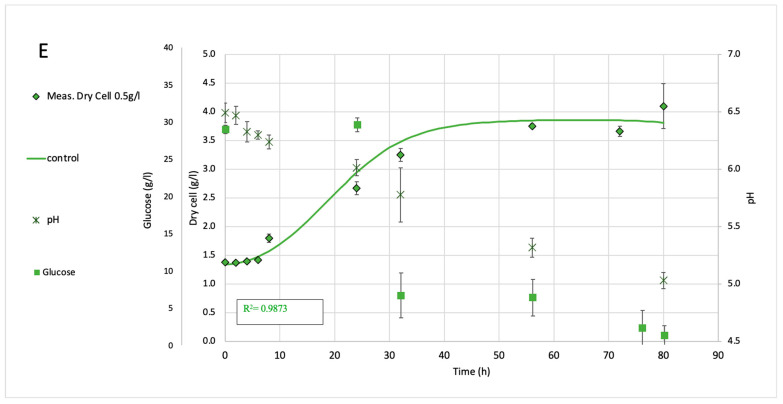
Fermentation of *B. licheniformis* with and without cyclodextrin supplementations: (**A**)—addition of a-CD (**B**)—addition of b-CD.; (**C**)—addition of g-CD; (**D**)—addition of DIMEB; (**E**)—control (without any CD).

*Bacillus licheniformis* was used in fermentation processes with varying a-, b-, and g-cyclodextrin (α-CD, b-CD, g-CD) and DIMEB concentrations to determine their influence on the pH (correlating with primary metabolites), dry cell (biomass) production, and glucose consumption. During the fermentation period, the pH values were monitored online, while the biomass production and glucose consumption were measured offline regularly via sampling. To analyze the growth dynamics, the OD_850_ values were fitted using a logistic (sigmoidal) regression model, which is appropriate for describing bacterial growth across the lag, exponential, and stationary phases. The product amount and effectivity (i.e., surface tension (ST) reduction) were determined only at the end of the fermentations. Figure 2 introduces the five types of biosurfactant fermentations: we established the growth curves under scaled-up flask conditions for all tested CDs, to confirm small scale (RTS) results with the exception of the specific growth rate. Umax could not be determined, due to the relatively rare manual sampling and (despite the high R^2^ values) to the fitted growth curves resulting in relatively high variance covering the effect of CDs.

Overall, the time-course profiles indicated that while β-CD and γ-CD had no effect on pH, biomass formation, or glucose consumption, the α-CD addition resulted in moderate concentration-dependent changes of biomass and glucose consumption with a steady pH. Interestingly, the DIMEB consistently enhanced both the biomass growth and glucose utilization with increased concentration, demonstrating its potential to improve the fermentation efficiency. Unfortunately, neither supplementation could overcome the control’s biomass amount, pH decrement, or glucose consumption.

### 2.3. Results of Surface Tension Measurments

**Figure 3:**
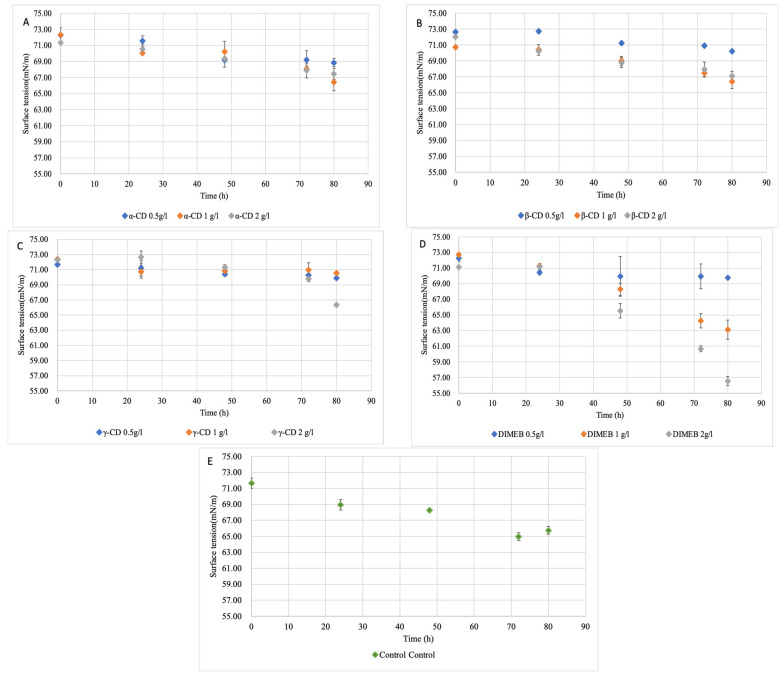
Surface tension results for cyclodextrin supplementations: (**A**)—addition of a-CD (**B**)—addition of b-CD.; (**C**)—addition of g-CD; (**D**)—addition of DIMEB; (**E**)—control (without any CD).

The time curves of the surface tension in all the fermentation tests are presented in Figure 3.

It was observed that, while neither a-CD, b-CD, nor g-CD could reach as low as 66 mN/m surface tension, in the case of DIMEB, two concentrations could go below this critical value defined by the control. This also showed that DIMEB seems to be an effective enhancing additive. To confirm, aside from the surface tension decrement effect, the biosurfactant was isolated from the fermentation broth via the acid-precipitation method.

Importantly, α-CD, β-CD, γ-CD, and DIMEB have been observed [33] to cause only negligible changes in the surface tension, typically remaining close to that of water (>70 mN/m); thus, the ST decrements observed in our fermentations were driven by microbial biosurfactant release.

### 2.4. Product Concentration

**Figure 4:**
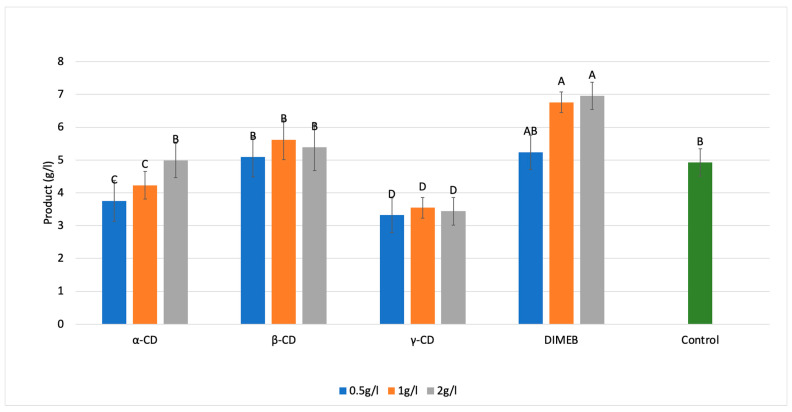
Biosurfactant yield results for different cyclodextrin types and concentrations compared to control group (green bar), Bars with different letters (A–D) indicate statistically significant differences between treatments.

The supplementation of different cyclodextrins (α-CD, β-CD, γ-CD, and DIMEB) at varying concentrations exerted distinct effects on the biosurfactant production by *Bacillus licheniformis*. The additives tested can be grouped into two groups: β-CD and γ-CD did not show any concentration dependency, but, independently from the applied dose, β-CD did not change the observed product amount compared to control, while γ-CD exhibited a significant reduction in the product amount. The second group of α-CD and DIMEB showed a concentration-dependent effect. However, while α-CD’s lower doses resulted in a lower product amount, the 2.0 g/L application reached the same as the control as shown in Figure 4. DIMEB, started at the same product amount as the control and higher doses, resulted in higher product values, suggesting that DIMEB is a good enhancer of lichenysin fermentative production.

Importantly, the added cyclodextrins did not precipitate during the downstream isolation process, suggesting they remained in solution and continued to act as inclusion complex formers without interfering with the product recovery [34,35]. Together, these results highlight that the type and concentration of cyclodextrins distinctly influence not just the fermentation kinetics but also the product yield, with DIMEB showing the highest potential as an effective additive to intensify the biosurfactant production.

Our results also confirmed that the surface tension measurement correlated with the extracted product amount; therefore, it can be used for small-scale experiments (for example, in small-scale high throughput development processes), where the samples’ volume is not enough to isolate measurable products via acid-precipitation.

To better understand the results and potential mechanism behind them, Table 2 summarizes the biomass increment (final–initial value) at 50 h (end of *trophophase*) and at 80 h (end of *idiophase*), the isolated product amount, the surface tension reduction (final–initial surface tension values), and the specific production yield (g product/g biomass). Regarding the biomass formation, similar tendencies were observed between the trophophase and idiophase except for the weaker results at α-CD and g-CD supplementation, as well as the absence of any enhancement detected in the trophophase. Regarding the product formation, b-CD consequently reached the same biosurfactant concentration and surface tension decrement like the control, indicating that it has almost no effect. At the same time, g-CD was permanently below the control’s results, while α-CD and DIMEB had strong concentration dependency: lower doses were farther from the control, but 2.0 g/L of α-CD reached the control; meanwhile, 2.0 g/L DIMEB even defeated the untreated runs with more than 41.43% regarding the product. The calculation of the specific production yield of the biomass resulted in a higher production yield in every CD treatment, which shed the light onto the usefulness of CDs in biosurfactant fermentations, since the biomass production capability could be increased by 10–80%, with highest specific production yield resulting from 2.0 g/L DIMEB supplementation.

To summarize from Table 2, we could observe that while b-CD was rather neutral to both biomass and product formation, g-CD was an inhibitor for both cell growth and product formation. At the same time, α-CD showed a special character: applying it in a small concentration, as 0.5–1 g/L, had a growth inhibition effect on product formation, but when applying at 2 g/L, it enhanced the biomass formation again. While DIMEB is a b-CD derivative, it showed a similar character to α-CD regarding a cell growth inhibitory effect at small concentrations (0.5–1 g/L) but a positive effect at 2.0 g/L for cell growth, with a remarkable enhancing effect for product formation. The highest reached product amount in the control was 4.96 ± 0.22 g/L licenysin, which is comparable to other authors’ results [36,37]. Meanwhile, our best production of 7 ± 0.17 g/L lichenysin observed with 2 g/L DIMEB supplementation was the highest.

## 3. Discussion

This study provides a detailed look at how different cyclodextrins and a special CD-derivative influence the fermentation performance of *Bacillus licheniformis*, focusing on growth kinetics, biomass accumulation, biosurfactant production, and surface tension activity. When trying to explain the experimental results, we considered that lichenysin is built by a five amino acid hydrophilic head moiety and a hydrophobic fatty acid chain. It has a very low critical micelle concentration (CMC), meaning that above 10 mg/L [11,38] lichenysin forms a micellar structure and does not remain alone in the solution. Furthermore, (1) CDs may have the capability to entrap some hydrophobic substrates or growth factors [39,40], (2) CDs may entrap the hydrophobic part of product lichenysin [41], (3) CDs may have a non-specific inhibition effect on the cell growth [42,43] and (4) CDs may also act on the product biosynthesis [44,45].

Considering the applied media recipe, substrate entrapment can be excluded, since only inorganic salts and glucose were used, which are neither hydrophobic nor large enough to form stabile inclusion complexes with any of the tested CDs or their derivatives.

Because of CDs’ molecule size, they cannot enter the cytoplasm, and thus, they cannot reach the biosynthetic enzymes of product formation; therefore, direct activation of product biosynthesis or direct inhibition can also be excluded.

Since b-CD had permanent neutral effect, it cannot contribute either negatively or positively to lichenysin fermentation, i.e., no interaction.

In contrast, g-CD has a permanent inhibitory effect on both cell growth and product formation, probably due to entrapping larger molecules, necessary for both phases of the process. Such molecules can be oligopeptides, which mediate between cells information on whether to grow or not such as the signal molecules of bacterial communications called Quorum Sensing (QS). For *B. licheniformis,* such a signal peptide was characterized as a 13 amino acid-formed oligopeptide ComX [46], whose size maybe appropriate to fit the largest cavity of g-CD, resulting in lower cell growth and lower product formation.

More interesting is the effect of α-CD, since in lower concentrations, it inhibited both cell growth and product formation, while in a higher concentration of 2.0 g/L, neither inhibition nor enhancement was detected. One could say that CDs can form in higher concentrations macro-structures (for example channel type, etc.), which cannot form inclusion complexes anymore [47,48]. This could explain why only lower doses resulted in inhibition. However, unfortunately, the known lowest amount to form such aggregates is around 10 g/L [49]; therefore, our 2.0 g/L seems to be not able to present a high enough surplus to form any type of aggregate. A possible mechanism leading to the presented results may be that, in the control experiments, the normally formed product lichenysin forms a micellar structure, which does not have any product inhibition effect in the tested concentration range. Applying 0.5–1 g/L of α-CD can destroy the micellar structure [50], through which product inhibition (probably via membrane-destroying) can be observed. Applying more α-CD (2.0 g/L) may also destroy the micellar structure of the product, but at the same time, there are free cavities to entrap, for example, the C15- hydrophobic tail of lichenysin, through which it can also decrease the membrane-destroying capability of the product, resulting no more inhibition. A similar mechanism can also be supposed for DIMEB taking also into account that methylation of b-CD narrows the cavity and also increases its hydrophobicity, capable of covering the tail of lichenisyn at a higher (2.0 g/L) concentration. The concentration-dependent effect of DIMEB is linked to its interaction with lichenysin micelles. Lichenysin, with its low CMC (~10 mg/L), readily forms micelles during fermentation; disruption of these aggregates at 1.0 g/L DIMEB may release free lichenysin monomers that increase membrane stress and suppress biomass growth. That mechanism partly eliminates the product inhibition (self-membrane-destroying), making it possible to produce 41% more lichenisyn [41,50].

The known size of CDs and the lichenysin tail can also support the above mechanism: the hydrophobic tail of lichenysin has a branch at its end; thus, the last carbon atom and its methyl groups size is similar to methane, of which the molecular diameter is 0.38 nm [51], while α-CD, β-CD, and γ-CD have ca. a 0.5, 0.6, and 0.75 nm cavity size, respectively. Methylation, according to Szente et.al. [52], does not change the cavity size, because hydroxyl groups of glucose are methylated in DIMEB at the outer part of the molecule; thus, the DIMEB shifted character from β-CD to α-CD is due to the increased hydrophobicity, which probably compensated the weaker bond in the inclusion complex.

Certainly, the above suggested hypothetical mechanism needs confirmation by further experiments; however, these results may help, even in their present form, to optimize the used CD amounts to find the CD concentration that can reach the highest product concentration without overdosing CDs, especially in such cases where oils are used for carbon sources in biosurfactant fermentation.

## 4. Materials and Methods

### 4.1. Bacterium Strain

*Bacillus* spp., *Bacillus licheniformis* DSM13, used in this research were obtained from the National Collection of Agricultural and Industrial Microorganisms (NCAIM, Budapest, Hungary) with the identification number B.02069T. To assure strain preservation, the bacteria were stored at 4 °C on Luria–Bertani (LB) medium-containing agar plates.

### 4.2. Cyclodextrin

The Alpha-cyclodextrin (α-CD), Beta-cyclodextrin (β-CD), Gamma-cyclodextrin (γ-CD), and Dimethyl-beta-cyclodextrin (DIMEB) used for this experiment were kind gifts from Cyclolab Ltd. (Budapest, Hungary).

### 4.3. Fermentation Process for Biosurfactant Production

To maintain strains, they were stored at 4 °C on Luria–Bertani (LB) agar plates (10.0 g/L tryptone, 5.0 g/L yeast extract, 10 g/L NaCl, 15.0 g/L agar). Fermentation experiments were conducted using two cultivation systems: the RTS-1 Personal Bioreactor (Biosan, Riga, Latvia) and Erlenmeyer shaking flasks.

While using the RTS-1 Personal Bioreactor to monitor the growth kinetics, cultures were incubated with a 10 mL working volume in a 50 mL Falcon tube and a 5% inoculation ratio. The system maintained rotation and temperature control, with the RTS rotating for 10 s in each direction at 250 rpm. The optical density at 850 nm (OD_850_) was measured automatically every 10 min. The specific growth rate (μ) was calculated from the exponential phase of the OD_850_ curve by its software.

For the shaking flask experiments, 150 mL of fermentation broth was dispensed into sterile 500 mL Erlenmeyer flasks. The flasks were sealed with cotton stoppers, wrapped in aluminum foil, and autoclaved at 121 °C for 30 min. Fermentation was initiated by inoculating with 5% (*v*/*v*) of a 24 h-old seed culture and incubated at 37 °C and 150 rpm in 250 mL Erlenmeyer flasks on 50 mL of the same defined media using a New Brunswick Excella E24 (McDonald Equipment, Hudson, MA, USA) shaking incubator for 80 h.

Both systems utilized the same defined minimal medium which consisted of 1 L of minimal media containing 34.0 g glucose-monohydrate, 6.0 g KH_2_PO_4_, 1.0 g NH_4_NO_3_, 2.7 g Na_2_HPO_4_, 0.1 g MgSO_4_*7H_2_O, 1.65 × 10^−3^ g FeSO_4_*7H_2_O, 2.2 × 10^−3^ g Na-EDTA, 1.2 × 10^−3^ g CaCl_2_, and 1.5 × 10^−3^ g MnSO_4_*4H_2_O. All chemicals (99% purity) used in this experiment were purchased from Reanal Laborvegyszer Kft., (Budapest, Hungary) [52,53]. To evaluate the effect of cyclodextrins on the microbial growth and biosurfactant production, both systems were supplemented with α-cyclodextrin (ACD), β-cyclodextrin (BCD), γ-cyclodextrin (GCD), and dimethyl-β-cyclodextrin (DIMEB) at concentrations of 0.5 g/L, 1.0 g/L, and 2.0 g/L, whose concentration range starts around the expected product concentration and decreases for feasibility. All experimental conditions, including the control (no cyclodextrin), were performed in independent triplicates.

### 4.4. Biomass Analysis

Monitoring bacterial growth in the fermentation broth was achieved by measuring the optical density at 600 nm with a CamSpec M501 Spectrophotometer (Spectronic CamSpec Ltd., Leeds, UK). The OD600 values were correlated with the dry cell mass, determined with a Sartorius MA35 moisture analyzer (Sartorius Lab Instruments GmbH & Co. KG, Göttingen, Germany) after the fermented broth was centrifuged to remove the cells, water was added to the pellets followed again with centrifugation to remove any media residue, and finally, the cell-pellet was dried and measured by the moisture analyzer. The OD600 values and corresponding dry cell weights for each bacterial replicate were plotted to generate a calibration curve, resulting in a high coefficient of determination (R^2^ = 0.9728). This curve was then used to estimate the biomass concentration in g cell dry weight/L according to the Equation (1) [53,54].

Biomass (g/l) = 0.4367 × OD_600_ + 1.3368 (R^2^ = 0.9728) (1)

For better visualization of shaking flasks cell growth, we fitted generalized the logistic equation (Equation (2)) to the measured data with Sigma Plot software (version.15) [55].

*x* = *xₘₐₓ*/[1 + *exp*(*a* + *b·t* + *c·t*^2^ + *d·t*^3^)] (2)

### 4.5. Glucose Analysis

For the analysis of glucose, isocratic high-performance liquid chromatography (HPLC) was utilized. A volume of 10 microL of appropriate diluted sample was injected into the Waters Breeze (Waters, Milford, MA, USA) HPLC apparatus, and 0.5 mL/min of mobile phase of 5 mM H_2_SO_4_ eluent (CARLO ERBA Reagents, Milan, Italy) flowed through the column. The study utilized a BioRad Aminex HPX-87H column (BioRad, Hercules, CA, USA) at 65 °C for efficient separation and analysis. Using a refractive index (RI) detector maintained at a constant temperature of 40 °C, compounds were detected [54].

### 4.6. Surface Tension

Using the stalagmometric technique and a Wilmad-Labglass LG-5050-100 stalagmometer (ATS Life Sciences, Wilmad LabGlass, Vineland, NJ, USA) (2.5 mL; tip: LG-5050-102), the surface tension of the liquid fermentation broth samples was determined. Measurements were performed in triplicate to ensure accuracy and reproducibility. The procedure followed the method described by Czinkóczky et al. [54]. Briefly, the number of drops formed by a fixed volume of each supernatant sample was counted, and the surface tension values were calculated relative to the distilled water, which was taken as the reference standard with an assumed surface tension of approximately 52 mN/m. This approach provided a reliable estimation of the relative surface tension changes resulting from biosurfactant production.

### 4.7. Isolation of Biosurfactant

To isolate the biosurfactant product and determine the manufactured product amount, the broth was centrifuged by Janetzki K23D (MLW, GDR) (Leipzig, Germany) at 6000 rpm for 30 min at 4 °C to separate the bacterium cells, obtaining a cell-free supernatant; the pH of the cell-free supernatant was adjusted by adding 6 N HCl, until it reached a pH of 2, measured continuously with FiveEasy, Mettler-Toledo (Greifensee Switzerland). After storing the acidified supernatant at 4 °C for 24 h to allow for the biosurfactant to precipitate, the biosurfactant precipitate was collected by centrifuging by Janetzki K23D (MLW, GDR) at 6000 rpm for 30 min at 4 °C. Using 6 N NaOH, the pH of the accumulated precipitate was adjusted to neutral. To obtain the purified biosurfactant, the sample was lyophilized using a Martin Christ Alpha 2-4 LSC lyophilizer, (Martin Christ Gefriertrocknungsanlagen GmbH, Osterode am Harz, Germany) which involved the removal of water. The quantity of biosurfactant obtained was then determined by weighing the lyophilized sample [53,56,57].

### 4.8. Statistics

Statistical analysis was performed using Sigma Plot (version 15, IBM, New York, NY, USA). Data were analyzed with one-way ANOVA (*p* < 0.05) and Tukey’s test to identify significant differences among groups.

## 5. Conclusions

The results seem to support the hypothesis that CDs, especially DIMEB, have the ability to increase *B. licheniformis* cell’s specific biosurfactant production yield (g product/g biomass). The effect of CDs on the biosurfactant production was found to be concentration-dependent in some cases. CDs altered the specific growth rate and the final product concentration differently. By encapsulating the hydrophobic fatty-acid tail of lichenysin, α-CD and especially DIMEB effectively reduces the product self-inhibition and lowers the culture surface tension, thereby boosting the biosurfactant yield without severely impairing the growth. Thus, the application of DIMEB is both beneficial for cell growth, but much more for lichenisyn production, and since lichenysin belongs to the most efficient biosurfactants, but its price limits its application, every cost-effective additive such as DIMEB, which enhances lichenisin’s formation, has a large impact. Since this is the first report on DIMEB’s biosurfactant production-improving effect, this underlines the novelty of our study. Future experiments should focus on confirming the presence and characterizing the entrapped biosurfactants, elucidating the specific mechanisms of cyclodextrins, using advance analytical techniques such as FTIR or HPLC-MS to enhance the biomass production or even confirming the suggested mechanism here and investigating cyclodextrins’ potential applications in various industries. In addition, further analytical techniques (like HPLC-MS or NMR) would be beneficial to confirm beyond any doubt the increased biosurfactant’s properties [57]. Furthermore, investigating the cytotoxicity and biocompatibility of CD-derived products will be critical for their safe and successful use.

## Figures and Tables

**Table 1:** Effect of cyclodextrin treatments on OD_850_ at 50 h compared to control.

Treatment	Concentration (g/L)	Mean OD_850_	Std Dev	*p*-Value vs. Control	Significance
Control	0	2.980	0.149	–	–
ACD	0.5	2.983	0.202	0.983	n.s.
ACD	1.0	2.940	0.142	0.753	n.s.
**ACD**	**2.0**	**3.787**	**0.463**	**0.045**	*
BCD	0.5	2.867	0.059	0.320	n.s.
BCD	1.0	3.243	0.881	0.658	n.s.
BCD	2.0	2.637	0.278	0.154	n.s.
GCD	0.5	2.857	0.827	0.822	n.s.
GCD	1.0	2.517	0.582	0.300	n.s.
GCD	2.0	2.823	1.104	0.830	n.s.
DIMEB	0.5	2.300	0.830	0.290	n.s.
**DIMEB**	**1.0**	**2.177**	**0.460**	**0.045**	*
DIMEB	2.0	3.497	0.638	0.294	n.s.

ACD—α-CD, BCD—b-CD, GCD—g-CD, n.s.—Not significant, * indicates significant difference compared to control.

**Table 2:** Comparison of the results of CD-supplemented and control fermentations for both trophophase and idiophase during *B. licheniformis* biosurfactant fermentation.

		Trophophase		Idiophase			
		Umax (1/h)	Biomass Increment RTS (g/L)	Biomass Increment in Flasks (g/L)	Product Conc. (g/L)	Surface Tension Decrement	Specific Product Formation Yield (g/g Cell)
α-CD	0.5 g/L	0.30 ± 0.05 ^b^	2.7 ± 0.16 ^a^	1.39 ± 0.04 ^b^	3.61 ± 0.14 ^b^	3.44 ± 1.16 ^b^	2.60 ± 0.17 ^b^
	1.0 g/L	0.29 ± 0.03 ^b^	2.69 ± 0.09 ^a^	2.09 ± 0.21 ^b^	4.34 ± 0.22 ^ab^	5.91 ± 2.14 ^a^	2.08 ± 0.13 ^ab^
	2.0 g/L	0.39 ± 0.03 ^a^	3.5 ± 0.32 ^c^	1.89 ± 0.03 ^b^	5.07 ± 0.1 ^ab^	3.9 ± 1.52 ^b^	2.68 ± 0.23 ^b^
b-CD	0.5 g/L	0.28 ± 0.02 ^b^	2.56 ± 0.02 ^a^	2.48 ± 0.16 ^a^	5.29 ± 0.22 ^a^	2.46 ± 0.66 ^b^	2.13 ± 0.07 ^ab^
	1.0 g/L	0.33 ± 0.01 ^b^	2.56 ± 0.02 ^a^	2.29 ± 0.41 ^a^	5.52 ± 0.11 ^a^	4.33 ± 0.63 ^b^	2.41 ± 0.16 ^b^
	2.0 g/L	0.32 ± 0.01 ^b^	2.56 ± 0.02 ^a^	2.31 ± 0.15 ^a^	5.42 ± 0.39 ^a^	4.92 ± 1.75 ^b^	2.35 ± 0.43 ^b^
g-CD	0.5 g/L	0.30 ± 0.04 ^b^	2.99 ± 0.10 ^c^	1.51 ± 0.14 ^b^	3.48 ± 0.15 ^b^	1.91 ± 0.89 ^b^	2.30 ± 0.23 ^b^
	1.0 g/L	0.29 ± 0.01 ^b^	2.54 ± 0.24 ^a^	1.73 ± 0.37 ^b^	3.54 ± 0.19 ^b^	1.83 ± 0.75 ^b^	2.05 ± 0.24 ^ab^
	2.0 g/L	0.28 ± 0.01 ^b^	2.48 ± 0.82 ^a^	1.39 ± 0.01 ^b^	3.26 ± 0.2 ^b^	5.95 ± 0.5 ^ab^	2.35 ± 0.45 ^b^
DIMEB	0.5 g/L	0.30 ± 0.05 ^b^	1.92 ± 0.58 ^b^	1.51 ± 0.23 ^b^	5.31 ± 0.2 ^c^	2.5 ± 1.15 ^b^	3.52 ± 0.14 ^c^
	1.0 g/L	0.29 ± 0.03 ^b^	1.92 ± 0.58 ^b^	2.07 ± 0.57 ^ab^	6.24 ± 0.64 ^c^	9.64 ± 0.31 ^c^	3.01 ± 0.55 ^c^
	2.0 g/L	0.35 ± 0.01 ^ab^	3.1 ± 0.36 ^c^	2.14 ± 0.56 ^ab^	7 ± 0.17 ^c^	14.59 ± 0.73 ^c^	3.27 ± 0.89 ^c^
control		0.39 ± 0.04 ^a^	2.55 ± 0.05 ^a^	2.72 ± 0.22 ^a^	4.96 ± 0.22 ^a^	5.9 ± 0.99 ^a^	1.82 ± 0.86 ^a^

White background color—no significant difference from the corresponding control value; green background—enhanced value compared to control; red background—reduced value to the control; a, b, and c in lower case represent the significantly differing groups.

## Data Availability

The data presented in this study are available on request from the corresponding author.
